# Brain network eigenmodes provide a robust and compact representation of the structural connectome in health and disease

**DOI:** 10.1371/journal.pcbi.1005550

**Published:** 2017-06-22

**Authors:** Maxwell B. Wang, Julia P. Owen, Pratik Mukherjee, Ashish Raj

**Affiliations:** 1 Department of Radiology & Biomedical Imaging, University of California, San Francisco, California, United States of America; 2 Department of Bioengineering & Therapeutic Sciences, University of California, San Francisco, California, United States of America; 3 Department of Radiology, Weill Cornell Medical College, New York, New York, United States of America; Oxford University, UNITED KINGDOM

## Abstract

Recent research has demonstrated the use of the structural connectome as a powerful tool to characterize the network architecture of the brain and potentially generate biomarkers for neurologic and psychiatric disorders. In particular, the anatomic embedding of the edges of the cerebral graph have been postulated to elucidate the relative importance of white matter tracts to the overall network connectivity, explaining the varying effects of localized white matter pathology on cognition and behavior. Here, we demonstrate the use of a linear diffusion model to quantify the impact of these perturbations on brain connectivity. We show that the eigenmodes governing the dynamics of this model are strongly conserved between healthy subjects regardless of cortical and sub-cortical parcellations, but show significant, interpretable deviations in improperly developed brains. More specifically, we investigated the effect of agenesis of the corpus callosum (AgCC), one of the most common brain malformations to identify differences in the effect of virtual corpus callosotomies and the neurodevelopmental disorder itself. These findings, including the strong correspondence between regions of highest importance from graph eigenmodes of network diffusion and nexus regions of white matter from edge density imaging, show converging evidence toward understanding the relationship between white matter anatomy and the structural connectome.

## Author summary

While the structural connectome of the brain has emerged as a powerful tool towards understanding the progression of neurologic and psychiatric disorders, links between the anatomy of connections within the brain and the effects of localized white matter pathology on cognition are still an active area of investigation. Here, we propose the use of the diffusion process towards understanding perturbations of brain connectivity. We find that while the dynamics of this process are strongly conserved in healthy subjects, they display significant, interpretable deviations in agenesis of the corpus callosum, one of the most common brain malformations. These findings, including the strong similarity between regions identified to be crucial towards diffusion and nexus regions of white matter from edge density imaging, show converging evidence towards understanding the relationship between white matter anatomy and the structural connectome.

## Introduction

The brain’s anatomic connectivity network or the “connectome” is the substrate upon which most of the brain’s complex phenomena are enacted, and through which various brain disorders ramify. It has recently emerged that the brain network can be decomposed into its constituent “eigenmodes”, which play a specific and important role in both healthy brain function and pathophysiology of disease [1–8]. The dynamics of any physical linear system can be described by a few constituent eigenmodes. The celebrated Fourier basis is a well-known example, where sinusoids of varying wavelengths are mathematically described as the eigenfunctions of any bounded-energy linear time-invariant filter [9]. Eigenfunctions are key features of classical mechanics, where for example, standing waves in continuous media are eigenfunctions. In quantum mechanics, the “probability cloud” of the electron’s orbit around the nucleus is described via eigenfunctions of the Schrodinger wave equation [10]. In structural biology, the so-called “normal modes” that describe the degrees of freedom of large molecules are the eigenfunctions of the equations that capture the relationship between the atoms of the molecule [11]. Similarly, many phenomena related to graphs or networks can be described in terms of the constituent graph eigenmodes, a field known as spectral graph theory [12].

In this paper we use spectral graph theory to obtain and characterize the brain’s organization through its eigenmodes. The brain graph consists of nodes that represent anatomically defined gray matter regions, and edges whose weights are given by white matter fiber connections deduced from fiber tractography. Here we show that the eigenmodes are predictors of brain phenomena by serving as mediators of networked spread processes within the brain. We had previously noted this role of the brain graph’s Laplacian matrix [13] eigenmodes in the context of brain activity propagation [2], and neurodegenerative pathology ramification [1]. Using network diffusion as the underlying model of spread, resting state BOLD functional connectivity (FC) was predicted in terms of structural network Laplacian eigenmodes [2]. A small number of Laplacian eigenmodes reproduced FC, and the eigenspectra of Laplacian and of FC are intimately related [14]. Other subsequent studies have explored the utility of the eigenmodes of the connectivity or adjacency matrix in capturing resting state functional networks [7, 8], in particular that a small number of such eigenmodes are sufficient to capture many elements of functional correlations in the brain [8].

The current study attempts to quantitatively characterize anatomic graph Laplacian eigenmodes of the human brain in order to cement the emerging understanding that Laplacian eigenmodes play a fundamental role in governing the spatiotemporal patterning of brain phenomena. We investigate to what extent a few low eigenmodes overlap between healthy subjects and between different scans of the same subject. We determine whether these eigenmodes are consistent across different atlas parcellations schemes. These results on consistency and conservation are necessary to support the role of Laplacian eigenmodes as substrates of information and pathology transmission within the brain. We next determine the influence specific white matter tracts exert on these eigenmodes, and show that these “importance maps” largely conform to previous maps [15, 16]. Finally, we explore eigenmodes in neurological disease where we show that agenesis of the corpus callosum results in eigenmodes that largely resemble intact brains, but with lower eigenvalues. In particular, we expect that the second eigenmode would capture information diffusion between the hemispheres, and would therefore be especially affected by callosal dysconnectivity. Taken together, the present study serves to formally characterize the brain’s eigenmodes in health and their behavior in disease.

## Methods

### Ethics statement

All study procedures were approved by the institutional review board at the University of California at San Francisco (UCSF) and are in accordance with the ethics standards of the Helsinki Declaration of 1975, as revised in 2008.

### Theory

We analyze the brain connectome, determined via diffusion-tensor imaging and probabilistic tractography, through analyzing the eigenmodes of the connectome’s network Laplacian. In summary, we investigate the neuroanatomical embedding of connections important to the Laplacian and how these constituent eigenmodes change in the case of agenesis of the corpus callosum.

#### Network diffusion model

In order to motivate the importance of Laplacian eigenmodes, we first show that they arise naturally from any simple linear first-order diffusion spread process on the graph, e.g. [1, 2]. Consider a brain network adjacency matrix, A, where we define the *i*, *j*-th indexed element of this adjacency matrix, $A_{ij}$, to represent the structural connection between the *i*-th and *j*-th cortical or subcortical gray matter structures. Here, we define this as the connectivity probability, determined via streamline count between the two structures, obtained from diffusion MRI data, as described later. Alternative methods, such as tract-tracing in animal studies, may also be used where available [17]. A diffusing entity *x*(*t*) on this graph will follow, in its simplest form, the so-called first order network-diffusion equation involving the graph Laplacian L [1].

$$\frac{dx}{dt}=\beta \left(\{\begin{array}{ll} -\sum_{k\neq i,j}^{}A_{ki} & i=j\\ A_{ij} & \text{otherwise} \end{array}\right)x\left(t\right)=-\beta Lx\left(t\right)$$ (1)

The above dynamics assume that the rate of transmission is a linear superposition involving pairwise differences, and ignores fast oscillatory behavior in the millisecond temporal scale. These assumptions are likely tenable only at the low frequency, macroscopic, whole brain level, which is the regime of interest in this paper. Thus, network diffusion is used here as a canonical example of network dynamics. Since Eq 1 involves the Laplacian matrix, its solution is accessible through the Laplacian’s eigendecomposition, where L is diagonalized as *Q*Λ*Q*^−1^. Here, *Q* is a square matrix whose columns are the eigenvectors of L and Λ is a diagonal matrix of the corresponding eigenvalues. This implies that the network dynamics can be interpreted as a linear superposition of eigenmodes. Since the eigenvalues are different and usually unique (disconnected graphs have repeating zero eigenvalues, and some regular graphs have repeating eigenvalues), some eigenmodes are more important than others. Specifically, it was postulated in [1] that only the slowest few eigenvectors are capable of capturing the most widespread and persistent patterns of spread. Indeed, the reciprocal eigenvalue 1/λ_*i*_ was shown to be related to the characteristic time of spread sustained by each eigenmode *i*, and as a result, the lower the eigenvalue, the slower the dynamics of the eigenmode is resolved. The slowest eigenmode is the rate-limiting one. Conversely, higher eigenmodes might sustain faster spreading and higher-frequency brain signaling; however, this aspect was not investigated here. A full exploration of higher eigenmodes will likely require more complete spread models that incorporate cortical processing and axonal conductance delays that play a critical role at high frequencies.

### Properties of healthy eigenmodes

#### Subjects and MR imaging

The sample used for this study consisted of ten healthy young adults (five male, five female; mean age 26.7 ± 5.9 years; nine right-handed) that were scanned twice with an average of 30.4 ± 2.7 days between scans. All study procedures were approved by the institutional review board at the University of California at San Francisco (UCSF) and are in accordance with the ethics standards of the Helsinki Declaration of 1975, as revised in 2008.

A 3 T TIM Trio MR scanner (Siemens, Erlangen, Germany) was used to perform MRI using a 32-channel phased-array radiofrequency head coil. High-resolution structural MRI of the brain was collecting using an axial 3D magnetization prepared rapid-acquisition gradient-echo (MPRAGE) T1-weighted sequence (echo time [TE] = 1.64 ms, repetition time [TR] = 2530 ms, TI = 1200 ms, flip angle of 7°) with a 256-mm field of view (FOV), and 160 1.0-mm contiguous partitions at a 256 × 256 matrix. Whole-brain diffusion-weighted images were collected at *b* = 1000s/mm^2^ with 30 directions.

The T1-weighted images were parcellated using the Desikan-Killiany atlas [18] into 66 cortical regions and 14 subcortical regions. Since the network Laplacian is highly sensitive to relatively isolated nodes (transmission from a nearly isolated node will diffuse extremely slowly into the rest of the brain, causing it to dominate persistent eigenmodes despite not being very physiologically relevant) we removed both the left and right frontal poles from the Desikan-Killiany atlas since the tractography algorithm used in this paper oftentimes registered only very weak connections between the frontal poles and the rest of the brain. The remaining 80 regions represented the nodes in each image’s corresponding brain network.

We also repeated this approach with the Destrieux atlas from Freesurfer and functional connectivity (FXCN) atlas to ensure that the results were not atlas specific [19, 20]. In the Destrieux atlas, three of 162 nodes were removed due to those nodes being disconnected: the right middle-posterior cingulate, the posterior-dorsal cingulate gyrus and the rectus gyrus. Four out of 347 nodes in the FXCN atlas were removed for the same reason.

Fiber estimation and tractography was performed as described in [15, 16]. In summary, Bedpostx was used to determine the orientation of brain fibers in conjunction with FLIRT [21]. In order to determine the elements of the adjacency matrix, $A_{ij}$, we performed tractography using *probtrackx2* [21]. More specifically, we initiated 4000 streamlines from each seed voxel corresponding to a cortical or subcortical gray matter structure and tracked how many of these streamlines reached a target gray matter structure. The weighted connection between these two structures, $A_{ij}$, was defined as the number of streamlines initiated by voxels in region *i* that reach any voxel within region *j*, normalized by the sum of the source and target region volumes ($A_{ij}=\frac{\text{streamlines}}{V_{i}+V_{j}}$). This normalization prevents large brain regions from having high connectivity simply due to having initiated or received many streamlines [22–24]. Afterwards, connection strengths are averaged between both directions (*A*_*ij*_ and *A*_*ji*_) to form undirected edges.

It is common in neuroimaging literature to threshold connectivity to remove weakly connected edges, as this can greatly influence the implied topology of the graph. In our work, we chose not to apply further thresholding, as unlike conventional graph theoretic metrics, linear models of spread and consequently network eigenmodes are relatively insensitive to implied topology induced by presence (or lack) of weak nonzero connections. However, to determine the geographic location of an edge, the top 95% of non-zero voxels by streamline count were computed for both edge directions. The consensus edge was defined as the union between both thresholded sets. These parameters were calculated in [25] to optimize test-retest reliability.

#### Lesion studies

We simulated the effect of virtual 12mm-diameter spherical lesions across the white matter regions of the brain to identify vulnerable regions. All edges passing through a lesion were weakened proportionally by the volume of the connection that was removed compared to the total volume of white matter occupied by the edge ($\Delta A_{ij}=\frac{\text{volume}\;\text{removed}}{\text{total}\;\text{edge}\;\text{volume}}A_{ij}$). This was done for two reasons. Firstly, while it would be ideal to instead block individual streamlines from tractography directly, due to limitations of the *probtrackx* software and computational limitations, this was not possible. Secondly, due to the relatively small fraction of tract volume that would be eliminated by a lesion, the perturbation upon the network structure and eigenmodes would be small. This is necessary to prevent the original and perturbed eigenmodes from representing completely different patters which would have made a quantitative comparison difficult. For reference, the angle between the original and perturbed eigenvector is roughly an order of magnitude less than the angle between the same eigenvectors of differing subjects (after subject to the “matching” scheme described below).

To explore the relationship between the power of the lesion and the found results, we simulated an additional experiment where the effect of a lesion on passing edges was calculated as described above, but then modulated further by a “stoppage” parameter which was varied as 25%, 50%, 75%, and 100%, where 100% would be the exact same original experiment. The “importance” of a white matter voxel with respect to a certain eigenmode was the resulting decrease in that eigenmode’s eigenvalue.

When generating the importance map for a single subject, we accounted for the potential “switching” of eigenvectors (what might be ranked as the fourth slowest eigenmode in one subject might be the fifth slowest eigenmode in another subject). For each scan, we selected the eigenmode that most resembles the averaged eigenmode of interest via an inner (dot) product and computed the importance map with respect to that selected eigenmode. Therefore, we calculated the inner product between the eigenmode of the averaged connectome and all eigenmodes of a given subject, and chose the best match. In practice, the “matched eigenmodes” were always within two steps away from each other. This difference occurs when in some subjects, the difference between successive eigenvalues is small enough to allow the eigenvector order to switch between neighboring eigenmodes. This does not represent a change in the eigenstructure itself which is demonstrated extensively in the Results where we show that the angle between these matched eigenmodes is not large enough to preclude their comparison.

#### Reproducibility

We tested the reproducibility of the found eigenmodes by measuring the average angle variance between eigenvectors. For each subject, we averaged the connectomes from both scans, creating a total of ten connectomes. These networks were then averaged and the resulting eigenmodes were calculated for the mean connectome. Each individual connectome’s angle variance for a given eigenmode was then defined as the angle between that connectome’s eigenmode and the corresponding eigenmode from the mean connectome. This angle was determined via dot-products of the relevant eigenvectors of dimensionality equal to the number of nodes in the atlas of interest. Note that in order to account for the potential “switching” of eigenvectors, we used the same procedure described in prior section to select eigenmodes for each individual scan. Additionally, since an eigenmode is fundamentally identical under sign reversal, we chose the sign conventions such that the angle variance would be minimized. This entire process is summarized in Eq 2, where ${\bar{v}}_{i}$ represents the mean eigenmode of interest and *v*_*j*_ represents the *j*-th individual eigenmode.

$$\sigma_{\theta ,i}=\min_{j}\left(\text{acos}\left|{\bar{v}}_{i}^{T}\cdot v_{j}\right|\right)$$ (2)

To create a baseline from which to compare these numbers to, we generated geometrically null random connectomes [26]. In summary, the Euclidean distance between the center of all pairs of nodes was calculated, and the strength of the connection between each node were sorted by this distance and organized into 100 discrete bins (bin sizes of 10 and 1000 were also tried with no significant change in results). All edges within each bin were then randomly shuffled with each other, generating random networks with similar cost-wiring relationships, allowing us to determine if the patterns in the network Laplacian are a function of the brain’s unique topography.

To assess the reliability of the dynamics of each eigenmode between subjects, we calculated the eigenvalues for each averaged subject connectome. Since we were interested in comparing the relative distributions of eigenvalues between subjects rather than their raw values, for a given subject, each eigenvalue was normalized by the mean of all eigenvalues associated with that subject prior to comparison.

We evaluated the reproducibility of the eigenmode importance maps using the concept of test-retest reliability through the intraclass correlation coefficient (ICC). ICC is defined as the ratio of intersubject variance to the sum of intersubject and intrasubject variance. This was found by averaging the importance of each white matter voxel across the 48 white matter tracts, as defined by the Johns Hopkins University (JHU) white matter atlas, for each individual scan and calculating the variance of these mean tract values between scans of the same patient versus between scans from different patients [27]. Defining the mean importance of JHU region *r* for subject *s* and scans 1 or 2 as *i*_*s*,*r*,1/2_, the ICC of region *r* is defined in Eqs 3 to 5.

$$\sigma_{1,r}=\mathrm{mean}\left(\text{Var}(\left\{i_{s,r,1},i_{s,r,2}\right\})\right)$$ (3)

$$\sigma_{2,r}=\text{Var}\left(\frac{i_{s,r,1}+i_{s,r,2}}{2}:s\in \left\{1,...,10\right\}\right)$$ (4)

$${\text{ICC}}_{r}=\frac{\sigma_{2,r}}{\sigma_{1,r}+\sigma_{2,r}}$$ (5)

#### Comparison of eigenmodes with rich club edge densities

The importance maps for each eigenmode averaged over all subjects and scans were compared to the averaged edge density maps for rich connections (RC), feeder connections (FC), and local connections as defined in [15, 16]. In summary, a rich club analysis, as defined in [28], was performed on the consensus connectome generated from the subjects used in this paper. Using the identified rich club network, each edge was split into three categories: connections between rich club nodes (RC), between rich club and non-rich club nodes (FC), and between non-rich club nodes (LC). Edge density maps were computed for each subject by identifying the geographic embedding of edges within the human connectome via tractography and identifying the number of edges passing through any given voxel. The resulting maps were then registered to MNI space using *flirt* and averaged over all subjects and scans [21, 29]. The mean and variance of the Pearson’s correlation coefficient between each eigenmode and edge density map was computed across all white matter voxels as defined by the Freesurfer parcellation. For reference, the correlation between importance maps from different eigenmodes was also computed.

### Agenesis of corpus callosum

#### Subjects and MR imaging

Seven individuals with complete AgCC and 11 healthy controls were prospectively enrolled. The two groups did not differ significantly (*p* > 0.05) with regard to age (AgCC: 24.3 ± 14.2 years; controls: 24.9 ± 9.1 years), gender (AgCC: 4 male; controls: 6 male), or Wechsler Adult Intelligence Scale full-scale IQ (AgCC: 102 ± 14; controls: 109 ± 17). All of the control subjects were right-handed, while the AgCC group was composed of five right-handed and two left-handed individuals. All study procedures were approved by the institutional review board at the University of California at San Francisco (UCSF) and are in accordance with the ethics standards of the Helsinki Declaration of 1975, as revised in 2008.

A 3 T MR scanner (GE Healthcare, Waukesha, WI) was used to perform MRI using a 8-channel head coil. Structural MRI of the brain was collecting using an axial 3D inversion recovery fast spoiled gradient echo T1-weighted sequence (TE = 1.5 msec, TR = 6.3 msec, inversion time 400msec, flip angle of 15°) with 230 mm FOV, 156 1.0-mm contiguous partitions at a 256 × 256 matrix. Preprocessing and tractography were performed as described in [30].

#### Comparison of eigenmodes between AgCC and virtual callosotomies

Other work has demonstrated the use of virtual callosotomies in identifying differences between AgCC and callosotomy structural connectomes [30]. Virtual callosotomies were calculated for each control subject by including an exclusion mask that would remove streamlines that passed through the corpus callosum when performing tractography. More specifically, a midline sagittal plane was manually drawn over the corpus callosum for each control subject. We then compared the eigenmodes and eigenspectrum of all three cases: healthy controls, AgCC subjects, and virtual callosotomies simulated on the controls.

## Results

Here, we describe the reliability of the slowest few eigenmodes of the network Laplacian, as well their neuroanatomical embedding in both gray and white matter. We compare these results to other methodology used in understanding the embedding of the brain graph, and categorize the use of the Laplacian towards understanding agenesis of the corpus callosum.

### Properties of healthy eigenmodes

#### Eigenmodes of the brain

**Fig 1:**
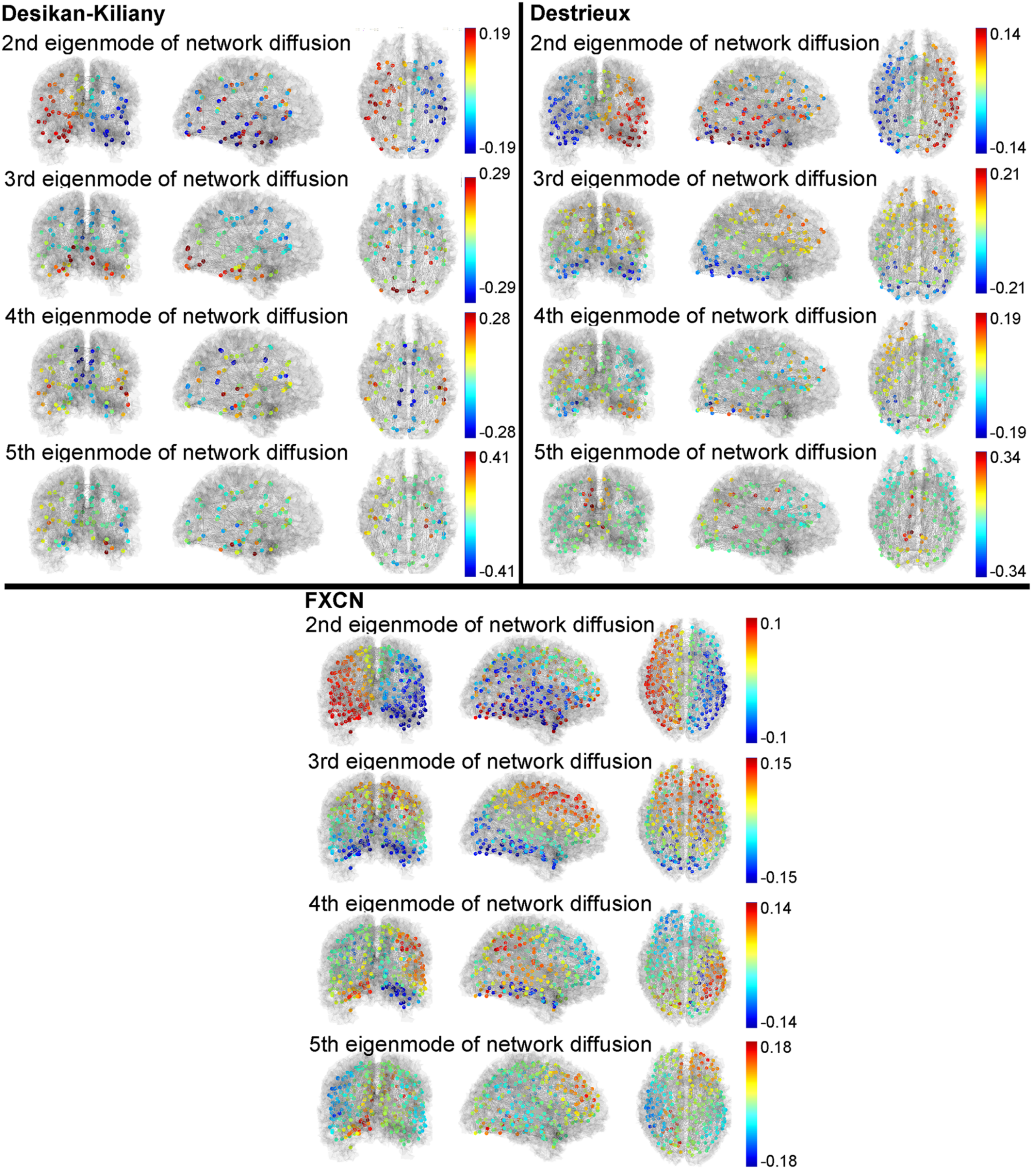
Eigenmodes of the network diffusion model: Brain network architecture determined by averaged connectomes from ten healthy adults (two scans each) using three different atlases: Desikan-Killiany, Destrieux, and functional (FXCN). We then computed the network Laplacian which models diffusion through the brain as shown in Eq 1. Colored dots represent the value of the eigenmode at any given node of the graph, both positive and negative, and are overlayed on top of a green, glass brain shown in the coronal, sagittal, and axial plane. These eigenmodes represent the four slowest diffusion processes in the brain network where diffusion occurs quickly between similarly colored nodes, and slowly from bright red to dark blue (or vice versa as eigenmodes are identical under sign reversal).

Fig 1 illustrates the slowest four non-trivial eigenmodes (sorted slowest to fastest) of the network Laplacian with the network analysis utilizing T-1 weighted MRI scans from ten healthy young adults, two scans each. Note that the first eigenmode is not shown as it is, by definition, constant across the entire network and represents the steady-state behavior of a system whose dynamics are governed by the network Laplacian. We find that each of the three slowest modes represent global transmission patterns of the structural connectome. More specifically, we find that they are diffusion between hemispheres, between superior and inferior areas, and between lateral and medial areas in the Desikan-Killiany atlas. These first two patterns are largely preserved across different gray matter parcellations while the latter two are not as consistent.

#### Importance maps

**Fig 2:**
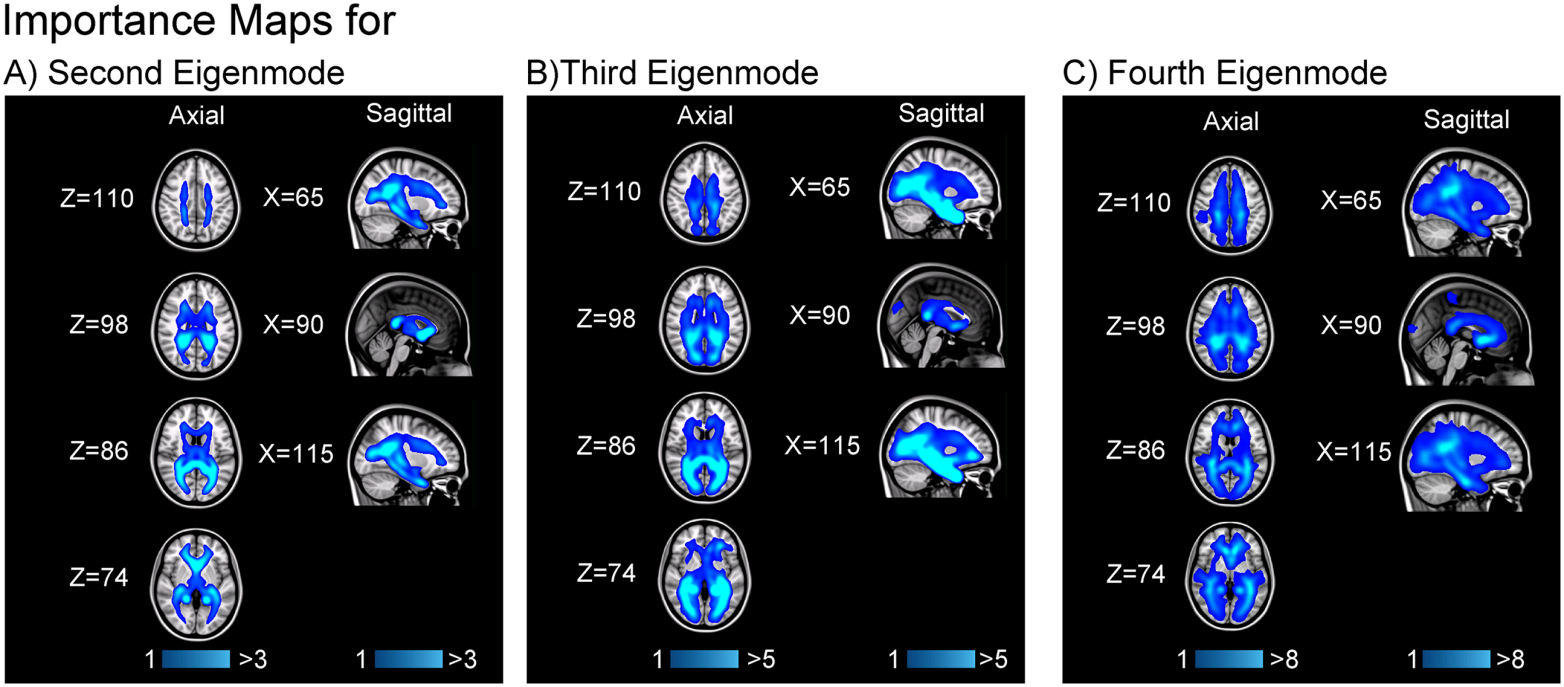
A white matter voxel’s importance for a given eigenmode is defined as the decrease in the corresponding eigenvalue when a 12mm-diameter lesion is placed at that voxel, damaging encompassed connections proportionally to the edge volume removed. These importance maps were averaged from the MNI152 1mm-registered maps of ten healthy adults, two scans each. The brighter the voxel, the larger the corresponding decrease in eigenvalue. The *X* and *Z* coordinates of each taken axial and sagittal plane are shown for reference: increasing *X* denotes the sagittal plane moving from the right to left side of the brain, while increasing *Z* denote inferior to superior axial planes.

Fig 2A–2C illustrate the mean importance map based on the decrease in the eigenvalue associated with the second, third, and fourth eigenmodes for a stoppage parameter of 100%. As we expect, since the second eigenmode represents spatial diffusion between hemispheres, the most vulnerable regions are in the corpus callosum, particularly within the genu and splenium. This pattern is repeated in the third and fourth eigenmodes. For the third eigenmode, we find that the highest vulnerability hotspots are in the periventricular areas. We also find that posterior tracts tend to be more important than their anterior counterparts. In the fourth eigenmode, we find the same weighting towards periventricular regions, although with less posterior bias. For reference, the average eigenvalue of λ_2,3,4_ are approximately 1300, 2600, and 3100 (unitless). Note that the percentage changes here are intentionally very small. While more aggressive lesioning methods would have generated larger percentages, they would also change the eigenmodes themselves dramatically making comparison between original and perturbed eigenmodes difficult.

**Fig 3:**
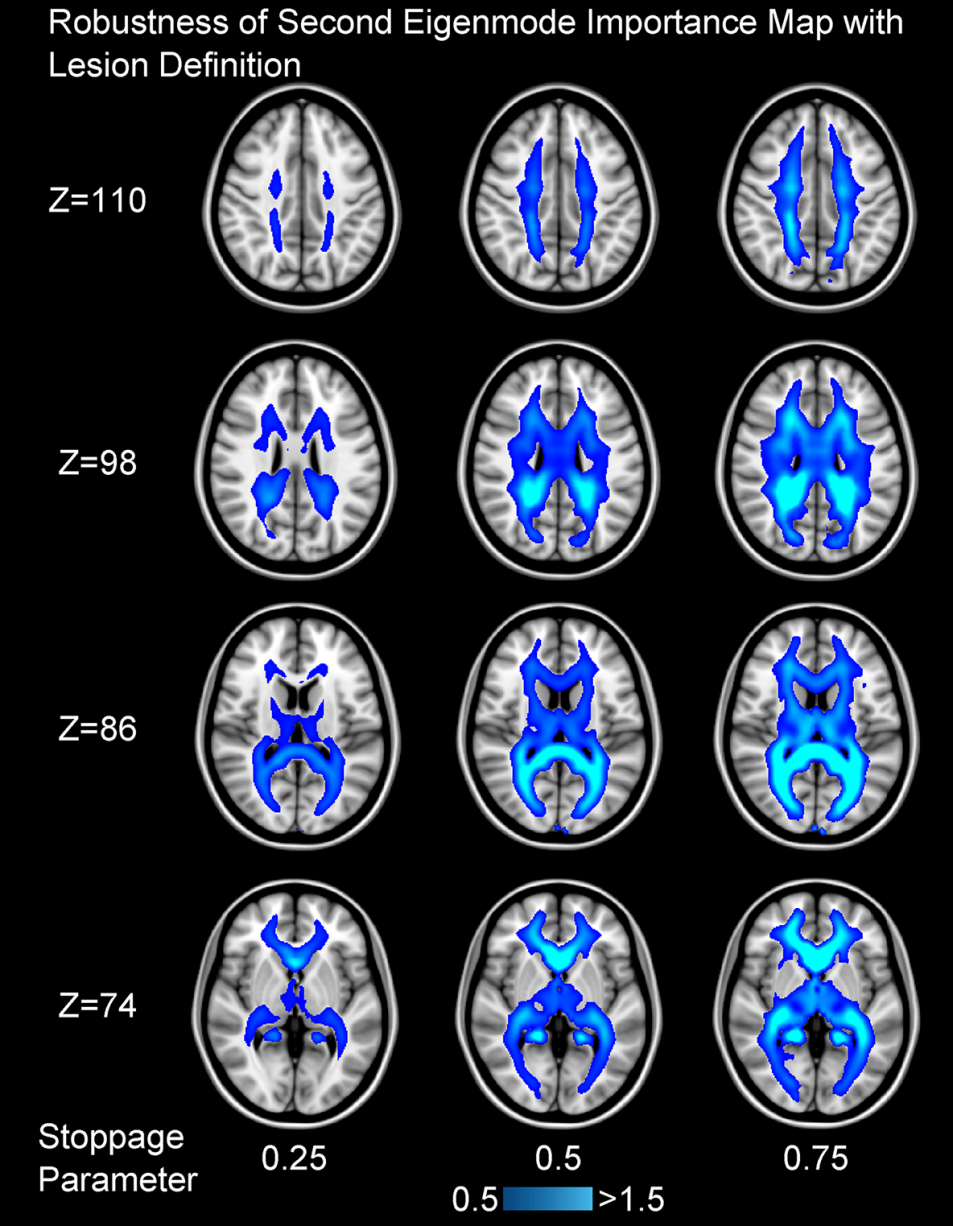
The procedure used to generate the importance maps shown in Fig 2 was repeated while testing the effect of partial lesions. Instead of simply weakening edges that pass through a lesion based on the volume of the edge removed, we modulated this edge loss by a “stoppage parameter” varying from (0,1) since it is possible for a region of damaged white matter to not fully stop streamlines from passing through it. Here, brighter colors represent that placing a lesion in that location caused a comparatively greater decrease in the second eigenvalue, weakening the rate of diffusion along the second eigenmode. Each column represents a different stoppage parameter, each row a different axial plane with the *Z* coordinate given in MNI152 1mm space. Here, increasing *Z* denote inferior to superior axial planes. For brevity, only the second eigenmode’s importance map is shown as the same geographical areas are highlighted regardless of this stoppage parameter, indicating that our analysis is robust and reliable under different interpretations of white matter damage.

As shown in Fig 3, we find that the importance maps for stoppage parameters of 25%, 50%, and 75% are very similar to the ones shown in Fig 2. After taking the Pearson correlation between all white matter voxels from averaged importance maps of the same eigenmode but differing stoppage parameters, we find that all r-values are bounded above 0.99 regardless of the eigenmode in question.

#### Reproducibility

**Fig 4:**
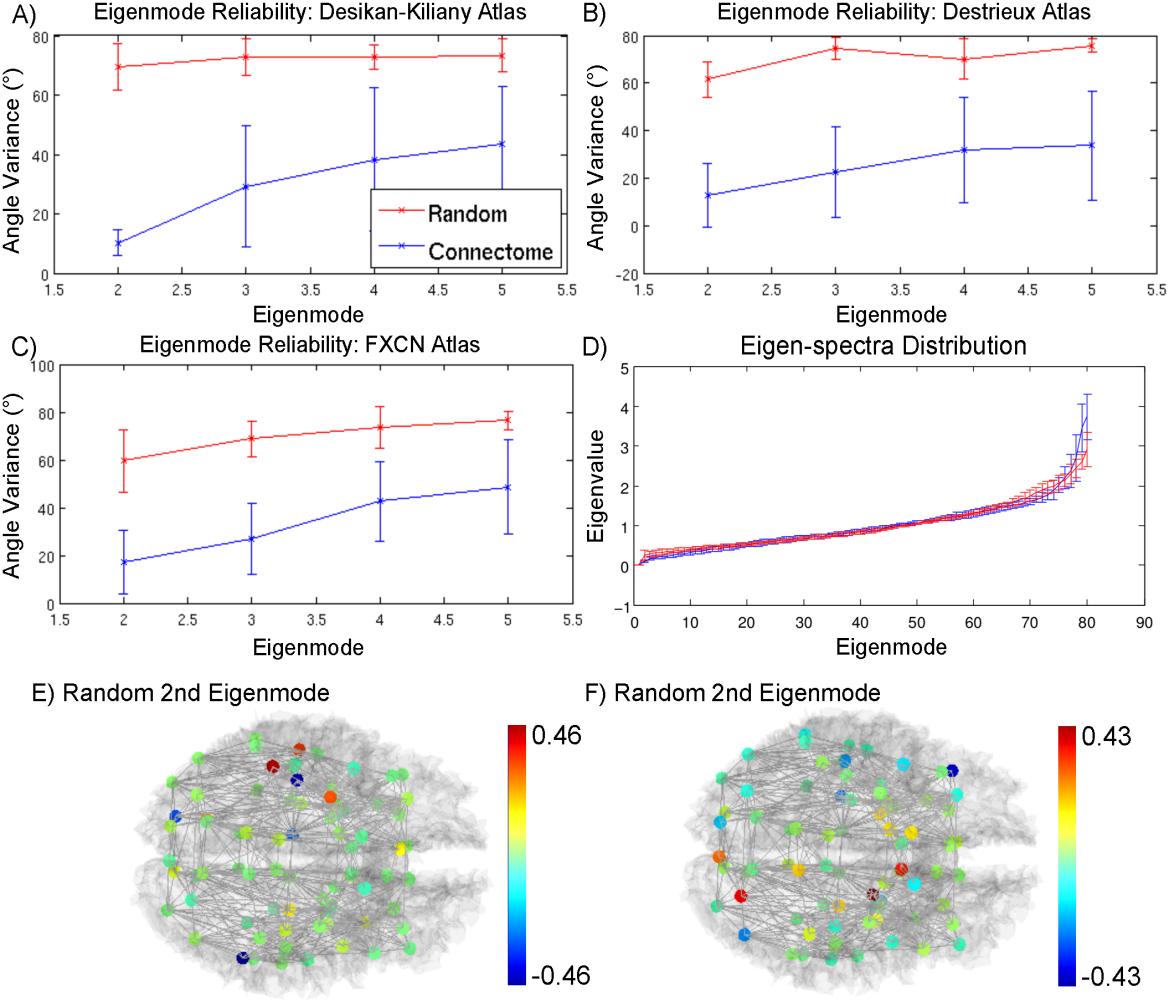
**A-C) The average angle variance of the eigenvectors from normal volunteers were calculated along with 95% confidence bounds and are shown in blue, with higher variance indicating less reliability in the eigenvector of interest.** For comparison, the average angle variance of eigenvectors for randomly generated networks are shown in red. Note that for the second and third eigenvector, the average angle variance is significantly lower than the average angle between eigenvectors from random networks, whereas the same does not hold true for the fourth eigenvector in the FXCN atlas. D) The distribution of the eigenvalues across all healthy subjects is shown in blue, with the distribution of eigenvalues in random networks shown in red. The eigenvalues of ten healthy adults were calculated individually using brain graphs averaged from two MRI scans from each subject and were then normalized by their mean. The resulting eigen-spectra were then averaged across all ten subjects and its 95% confidence interval was plotted as error bars. Eigenmodes are numbered in increasing order of eigenvalues. Insets for the lowest and highest eigenmodes were added for clarity. Statistical significance marked as * was determined via two-sample t-tests with an *α* of 0.05. E-F) The second eigenmode for two randomly generated brains are shown for comparison.

Fig 4A–4C shows the average angle variances between eigenmodes of different subjects for both the actual brain connectome and randomly generated geometric-null networks with 95% confidence bounds. We find that the brain connectome’s variance in the second through fifth eigenmode in all atlases fall below the variance of the geometrically-null network in all atlases. Fig 4D similarly shows that the eigen-spectrum of both the brain connectome and random network is relatively conserved for the intermediate range of eigenmodes. As shown by the insets of the lowest and highest eigenmodes, while the first eigenvalue is guaranteed to be zero, the lowest non-zero eigenvalues for the human connectome are lower than those of the geometrically-null network, the second eigenvalue being statistically significantly different. This pattern is reversed for the higest few eigenvalues. For visual comparison, the second eigenmodes for two of the randomly generated networks are shown in Fig 4E and 4F to indicate the lack of a consistent structure unlike the second eigenmodes of healthy controls.

Since two scans were collected for every subject, we also calculated the within-subject angle variance and found that it was not statistically significantly different from the variances shown in Fig 4. This is likely an issue with sample size as each subject has one within-subject comparison to make as opposed to nine other between-subject comparisons.

**Fig 5:**
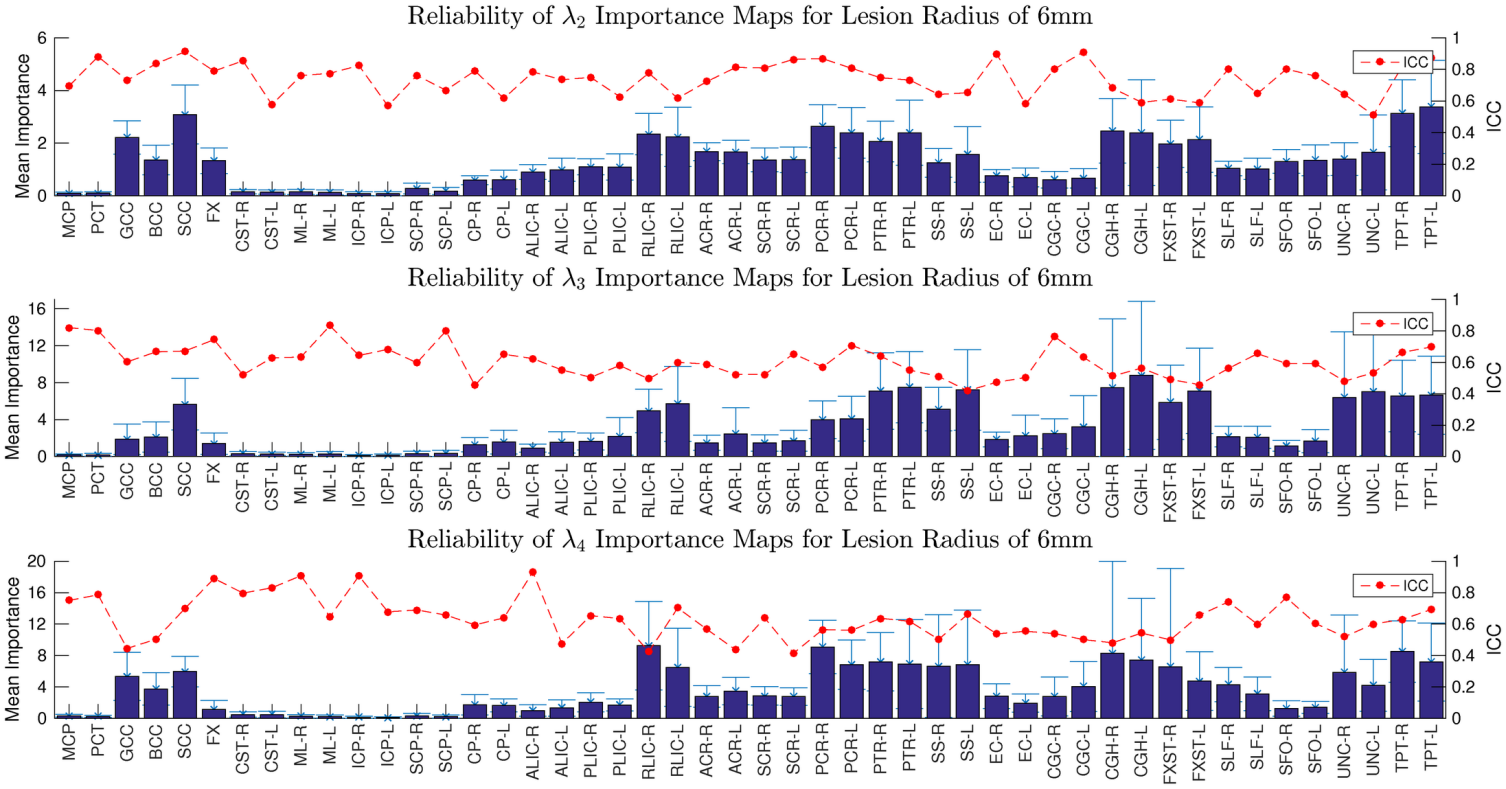
Reliability of mean importance for the second, third, and fourth eigenmodes. The heights of the bars show the mean importance in the white matter tract of interest and the error-bars show the standard deviation. The red points indicate ICC. The importance of a white matter voxel with respect to an eigenmode is defined as the decrease in the corresponding eigenvalue when a lesion of diameter 12 millimeters, centered on the voxel of interest, is cut, damaging all connections that pass through the lesion proportionally to the volume of the edge removed by the lesion. Here the labels on the x-axis are the abbreviations for the white matter tracts defined in the Johns Hopkins University (JHU) DTI-based white matter atlas [27].

The test-retest reliability of the importance maps across the 48 JHU white matter tracts is shown in Fig 5 [27]. While the ICCs of the importance maps for the second eigenmode are bounded above 0.5, they occasionally decrease to approximately 0.4 for the fourth eigenmode, indicating the decreasing reliability of higher eigenmodes. According to well-established clinical guidelines, ICC values below 0.4 are considered poor reproducibility, ICC values between 0.4 and 0.75 are considered fair-to-good reproducibility, and ICC values above 0.75 are considered excellent reproducibility [31, 32].

Note that the distribution of mean importance is relatively similar between all three eigenmodes. As these lowest eigenmodes represent global patterns, the core of tracts in the center of the connectome that are responsible for mediating large-scale transmission, such as the corpus callosum, would be particularly vital to these modes when compared to tracts on periphery of the network. Here, we hypothesize that this core could be possibly related to the major hubs of the brain network and sought to test this relationship through analyzing edges involved with the rich club [28].

#### Comparison of eigenmodes with rich club edge densities

In order to assess the similarity of the eigenmode importance maps proposed in this paper, we sought to compare them to other metrics of tract importance to overall neural connectivity. As we are particularly interested in the embedding of these eigenmodes in white matter, we utilized the edge density maps from [15, 16]. Each edge density map (rich club, feeder, and local connections) represent the density of white matter connections as defined by tractography throughout the brain and were calculated on the same subject group used in this paper (ten healthy adults with two scans each).

**Fig 6:**
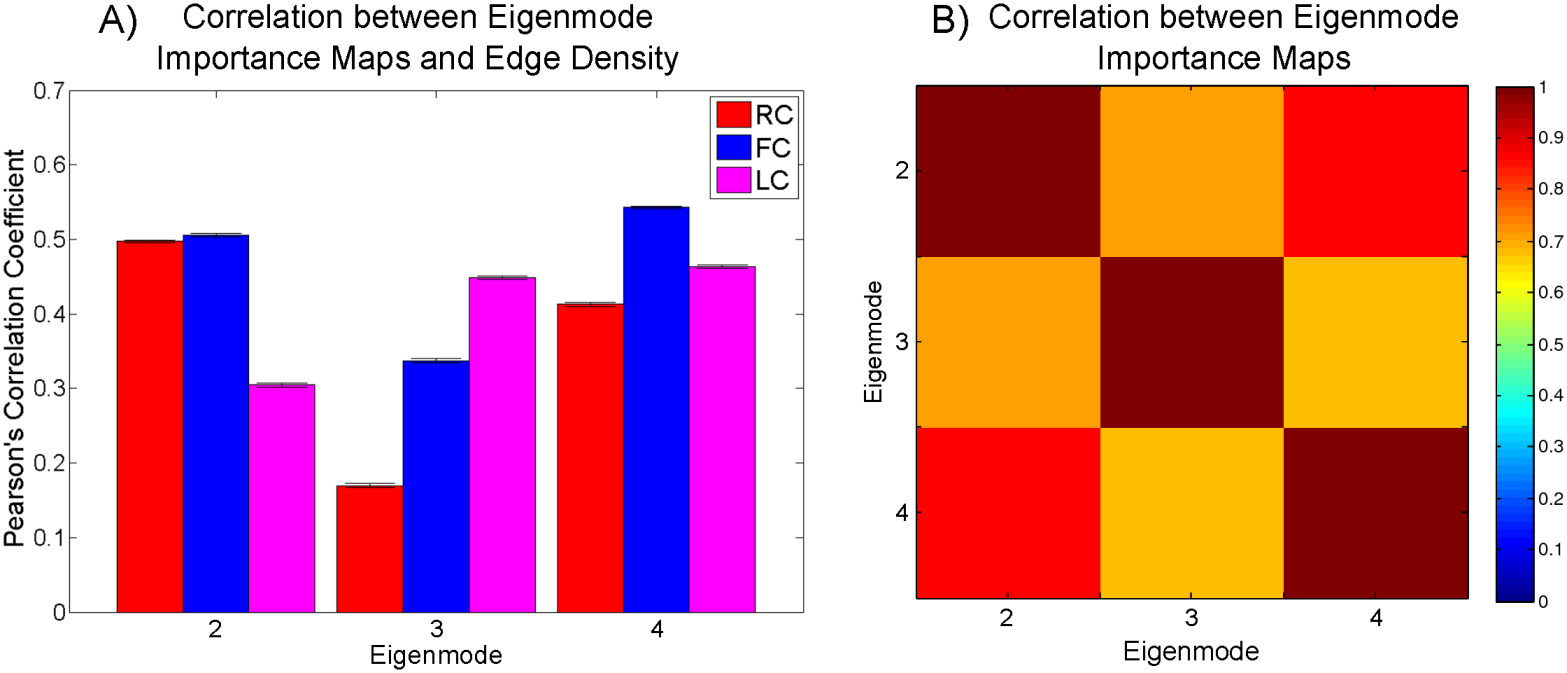
**A) The Pearson’s correlation coefficient was calculated between the eigenmode importance maps and edge density maps from [15, 16].** Each edge density map (rich club, feeder, and local connections) represent the density of white matter connections throughout the brain and were calculated on the same group of healthy adult subjects used to generate the averaged importance maps shown in Fig 2. Each edge density map was then averaged across all subject and then the Pearson correlation of white matter voxels was taken between each density map and the averaged eigenmode importance maps in Fig 2. The error bars reflect the 95% confidence intervals of the Pearson’s correlation coefficient. B) The Pearson’s correlation coefficient was calculated between each averaged eigenmode importance map and shown as a heatmap where brighter squares indicate a higher correlation between the pair of eigenmodes shown on the *x* and *y* axes.

Fig 6A illustrates the Pearson’s correlation coefficient between eigenmode importance maps and edge density maps along with confidence bounds. We find that rich club connections show high similarity to the second eigenmode’s importance map, while local connections are most similar to the third eigenmode. While the fourth eigenmode displays correlation with all three edge density maps, it’s lack of statistical reproducibility makes its interpretation much less reliable.

In Fig 6B, we find that all three importance maps are highly correlated with each other despite the large correlation differences in Fig 6A. This correlation is likely driven by the high importance given to posterior periventricular white matter which is consistent with observations in [15, 16]. One possible reason for the particularly high correlation between the second and fourth eigenmode importance map is that both modes share many of the same white matter tracts with the primary difference being that the second eigenmode has a higher representation of rich club edges whereas the fourth eigenmode has a higher representation of local edges.

Of particular interest is the second and third eigenmode, which despite their strong correlation to each other, show large differences in their correlations to rich and local club edge densities. This indicates that the slowest diffusion processes rely disproportionately upon a subnetwork of nodes with high degree when compared to the third, faster, eigenmode. This is not immediately apparent as “rich club” and Laplacian eigenmodes are defined in different contexts and represent converging evidence towards the influence of these white matter tracts on connectome architecture.

### Agenesis of corpus callosum

**Fig 7:**
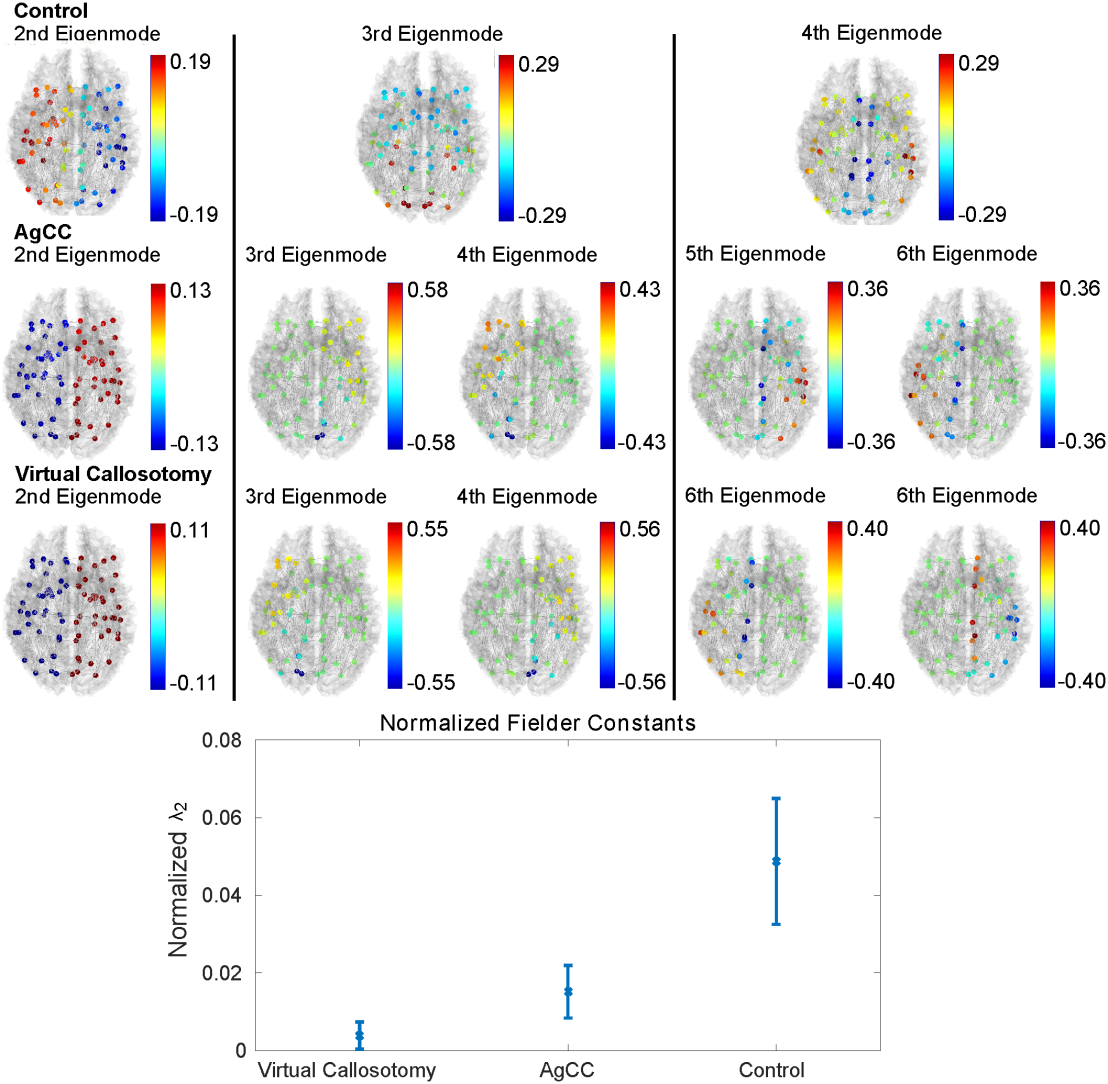
Effect of corpus callosum on connectome eigenmodes. **Top) brain network architecture determined by averaged connectomes from eleven healthy adults for the control, seven cases of complete agenesis of corpus callosum (AgCC), and the same eleven healthy adults with virtual callosotomies.** Note how the second eigenmode in all three cases represents diffusion between both hemispheres, albeit more polarized in cases of AgCC and virtual callosotomies, due to the lack of an intact corpus callosum. For higher eigenmodes, we see that each eigenmode in the control group is split into two separate modes in the AgCC or virtual callosotomy cases, one for each hemisphere. Intuitively, the corpus callosum synchronizes diffusion across both hemispheres, and its absence allows transmission to occur within one hemisphere with no effect on the other which is reflected in the changed eigenmodes. Bottom) the eigenvalue corresponding to the speed of diffusion in the second eigenmode (left-right communication) is shown along with 95% confidence bounds. All differences in eigenvalues are statistically significant.

Fig 7 (top) illustrates the first three eigenmodes of the control group and their corresponding eigenmodes in agenesis of corpus callosum (AgCC) and virtual callosotomy subjects. In all three cases, the second eigenmode represents diffusion between both hemispheres, albeit more polarized in cases of AgCC and virtual callosotomies, likely due to the lack of an intact corpus callosum. Instead of the gradual diffusion we see in healthy brains from one lateral side to medial areas of the brain and finally to the opposite lateral side, the bottleneck in left-right diffusion occurs solely at the corpus callosum in AgCC and virtual callosotomies. For higher eigenmodes, we see that each eigenmode in the control group is split into two eigenmodes in the AgCC or virtual callosotomy cases, each eigenmode representing the original control eigenmode across only a single hemisphere.

Fig 7 (bottom) shows the eigenvalue associated with the second eigenmode, left-right diffusion, normalized by the mean eigenvalue. We find that in cases of AgCC, the normalized second eigenvalue is statistically significantly (*p* < 0.05) higher in cases of AgCC than it is in virtual callosotomies, indicating a faster rate of diffusion between hemispheres. We also find that both AgCC and virtual callosotomies show lower normalized second eigenvalues than the control group.

## Discussion

The brain network is a “small world” [33], has highly efficient communication capacity [34] and is highly modular, with some structures acting as hubs and some are critical for connecting different modules [33]. Hub regions, in turn, preferentially connect to other hubs, forming a so-called “rich club” [28]that appears to anchor a “structural core” [35]. These properties come at some of the smallest wiring costs [36]. Several graph metrics are available to characterize how mutual dynamic influences or perturbations can spread within the underlying structural brain network, e.g. communicability, defined as the number of paths between nodes weighted by the path length [37], and analytic measures like search information and path transitivity [34, 38]. While these graph theoretic metrics give a rich phenomenological and statistical description of brain networks, a parsimonious, low-dimensional descriptor is missing.

Here, we presented evidence that the eigen-decomposition of the Laplacian provides a parsimonious basis and an important addition to existing graph theory metrics, a concept that naturally arises from linear models of network transmission. The graph Laplacian is a discrete analog of the continuous Laplace operator Δ^2^, hence it naturally captures the process of graph diffusion [1, 2, 39]. We had previously proposed that the brain graph’s low eigenmodes act as network attractors for neurodegenerative pathologies [1], brain activity [2, 14] and the spread of hyperactivity in epilepsy [3]. Since these eigenmodes have the lowest eigenvalues, linear dynamics will settle into a few of these modes which are found to be highly consistent between subjects. Only a small number of constituent eigenmodes of the structural Laplacian were sufficient to reproduce BOLD-derived functional connectivity patterns [14].

We note that some of these theoretical results are by no means novel to the current paper or to our group. A vast body of related work exists in computer science and to a more limited extent in neuroscience as well. The relationship between SC eigenvectors and FC was previously suggested in the local circuit context [40]. A few anatomic Laplacian eigenmodes exhibited an uncanny resemblance to the spatial templates of BOLD resting state networks [8]. Eigen-spectra of the Laplacian was found to be relatively conserved across various species [41]. There is a strong correspondence between Laplacian eigenmodes and the graph theoretic concept of modules; spectral graph theory relies on Laplacian eigenmodes to compute strongly connected node clusters, e.g. normalized cuts [42]. Indeed, the spectral clustering algorithm of [42], when applied to the brain graph, yielded modules with a hierarchical base-2 tree architecture, where modules appeared to fall out from the eigenmodes of the graph [43]. For instance, the use of eigenmodes towards modeling wave propagation under neural field theory has been reviewed elsewhere [7]. The view of brain activity as a linear superposition of eigenmodes evokes Nunez’s conception of global EEG rhythms as spatial eigenmodes [44–46]. A full survey of this body of work is infeasible here due to limited space, and our key point is to showcase that Laplacian eigenmodes can have more widespread applicability in brain science than commonly thought.

Of course, many brain phenomena can be accessed only using non-linear models, for instance cascading behavior resulting from cooperative and competitive dynamics [47]. Interestingly, some of these models, such as neural field theory, take advantage of the same operators, namely the network Laplacian, making the study of these operators highly relevant [7]. While local brain dynamics are not linear or stationary, the emergent stationary behavior of long-range interactions can be independent of detailed local dynamics [34], and ensemble-averaged behavior of large connected but individually nonlinear neural populations can be quite linear [48].

### Low Laplacian eigenmodes appear to be conserved between individuals

First we showed that the network Laplacian possesses largely conserved eigenspectra (Fig 4) between subjects. The eigenvalues associated with the eigenmodes, i.e. the eigenspectrum, provide a natural ordering of the eigenvectors. As argued previously, the reciprocal eigenvalues $\frac{1}{\lambda_{i}}$ represent characteristic time constants of diffusive processes within each eigenmode *i*. Hence the long-term patterns of any linear process of spread, whether of activity or pathology, will essentially settle into the eigenmodes with the lowest eigenvalues. We found that these first few eigenmodes of a subject are not overly sensitive to atlas parcellation and connectome extraction techniques (Fig 1). Further, the lowest eigenmodes are reasonably stable, reproducible and consistent within and amongst subjects (Figs 4 and 5). These results serve to confirm that spectral graph theory of brain processes will likewise be generalizable within, between and across healthy subjects, suggesting that low eigenmodes might represent some invariant properties of brain networks. Why should graph eigenmodes in one person’s network be similar to another’s at all? First, healthy subjects show canonical connectivity patterns at the macroscopic level despite significant variability in specific tracts and regions. Perhaps as a consequence, they are able to sustain highly repeatable functional connectivity patterns, e.g. the default mode network [49]. While we were able to rule out the effect of spatial cost-wiring rules through the use of geometrically-null random networks, there are still many conserved properties of brain networks, such as small-world and rich club properties, that can contribute to this consistency. Second, low eigenmodes may be conserved between individual brain networks, and only higher eigenmodes might be responsible for inter-subject variability. This is not unlike the low frequency harmonics displayed by many simple physical systems of diverse origin. It would be interesting to explore in more detail the topological factors that are driving the invariance in the brain’s low eigenmodes, and to test whether certain wiring rules also produce these invariants. This is a subject of future work.

### Relationship to hubs and critical networks

Previous studies on the the effect of lesions on the brain connectome largely focus on the node level, i.e. gray matter regions. In particular, rich club nodes have been implicated in neuropsychiatric disorders like schizophrenia, and Alzheimer’s disease [28, 50]. Here we used eigenmodes to explore edge-level effects of lesions. While other approaches have identified white matter areas of high importance by examining the effects of simulated lesions on network performance measures such as efficiency or characteristic path length [51], these maps often denote lateralized focal peripheries as the areas of highest importance and show little overlap with the rich club connections from [28] and [15, 16]. We believe that eigenmodes provide an alternative approach. By separating overall transmission within the brain into its slowest, individual rate limiting eigenvectors, we can assess which white matter voxels and edges are most important to them. Interestingly, we find that importance maps for the second and fourth eigenmodes showed similarities to edge density maps of rich club and feeder connections, e.g. [28], while the third eigenmode is similar to local connections (those between non-rich club nodes). These similarities are robust under changes to how the lesion is defined, indicating they are latent properties of the network. In all cases, we find heavy weighting within posterior periventricular white matter, mirroring the observations in [15, 16]. These similarities are interesting because “rich club” and Laplacian eigenmodes were historically defined in completely different contexts. While rich club nodes are associated with high degree by definition, it is not immediately apparent that the slowest diffusion processes would rely on this subnetwork disproportionately when compared to the third, faster, eigenmode. Possibly, faster diffusion processes rely on a broader range of connections, both rich and non-rich, when compared to the second eigenmode, and these redundancies de-emphasize rich club edges. Regardless, the convergence of the eigenmode- and edge density-derived importance maps from [15, 16] is a promising indicator of the relationship between white matter anatomy and the structural connectome.

### Eigenmodes of impaired brains

Given that healthy brain eigenmodes appear largely conserved between and within subjects, next we explored how they might change in impaired brains. An intuitive way to investigate the influence of the corpus callosum, the largest inter-hemispheric fiber bundle, is through the study of individuals who are born without it, a condition known as agenesis of the corpus callosum (AgCC) [52]. Interestingly, these individuals often outperform patients with a corpus callosotomy on various motor control tasks that required inter-hemispheric communication [53]. Past studies on AgCC have focused largely on analysis of resting state fMRI networks [54, 55]. Many studies note that AgCC subjects tend to perform closer to healthy subjects compared to patients who underwent corpus callosotomies and ascribe this difference on “replacement” tracts that are only present in AgCC cases, presumbably via adaptation [30]. In this paper, we were able to provide a quantitative intuition to the effect these structural adaptations have on inter-hemispheric communication through the network Laplacian. Specifically, we found statistically significant differences between the eigenspectra of AgCC and virtual callosotomy brains. Additionally, we validated the intuition that the low healthy eigenmodes might “split” into separate eigenmodes in cases of virtual callosotomies and AgCC, due to lack of inter-hemispheric connections. Many theoretical results exist on the eigenspectra of disjoint graphs [13], which predict exactly this behavior.

These results taken together serve to quantitatively characterize the anatomic graph Laplacian eigenmodes of the human brain in order to cement the emerging understanding that Laplacian eigenmodes play a fundamental role in governing the spatiotemporal patterning of brain phenomena. We find that these eigenmodes are consistent and robust across different atlas parcellations schemes, and also determine the influence specific white matter tracts exert on these eigenmodes. Finally, we explore eigenmodes in neurological disease where we show that agenesis of the corpus callosum results in eigenmodes that largely resemble intact brains, but with lower eigenvalues. In particular, we expect that the second eigenmode would capture information diffusion between the hemispheres, and would therefore be especially affected by callosal dysconnectivity. Together, the present study serves to formally characterize the brain’s eigenmodes in health and their behavior in disease.
